# Mitochondria, Peroxisomes and Beyond—How Dual Targeting Regulates Organelle Tethering

**DOI:** 10.1177/25152564241264254

**Published:** 2024-09-28

**Authors:** Johannes Freitag, Thorsten Stehlik, Gert Bange

**Affiliations:** 1 Center for Synthetic Microbiology (SYNMIKRO), 9377 Philipps-University Marburg, Marburg, Germany; 2 Department of Biology, 9377 Philipps-University Marburg, Marburg, Germany; 3 Department of Chemistry, 9377 Philipps-University Marburg, Marburg, Germany; 4 Molecular Physiology of Microbes, Max-Planck-Institute for Terrestrial Microbiology, Marburg, Germany

**Keywords:** dual targeting, protein trafficking, tether, peroxisome, mitochondrion, targeting signal

## Abstract

Eukaryotic cells feature distinct membrane-enclosed organelles such as mitochondria and peroxisomes, each playing vital roles in cellular function and organization. These organelles are linked at membrane contact sites, facilitating interorganellar molecule and ion exchange. Most contact-forming proteins identified to date are membrane proteins or membrane-associated proteins, which can form very stable contacts. Recent findings suggest additional mechanistically distinct tethering events that arise from dual protein targeting. Proteins bearing targeting signals for multiple organelles, such as an *N*-terminal signal for mitochondria and a *C*-terminal signal for peroxisomes, function as tethers, fostering contacts by engaging targeting factors at both organelles. A number of dually targeted membrane proteins can contribute to contact site formation and transit from one organelle to the other as well. These interactions may enable the fine-tuning of organelle proximity, hence, adapting connections to meet varying physiological demands.

## Dual Protein Targeting: Proteins With More Than One “Zip Code”

It is widely recognized that proteins assigned for specific membrane-bound organelles harbor targeting signals similar to zip codes, which are recognized by organelle-specific targeting factors either during or after translation in the cytosol (Blobel and Sabatini, 1971; Wickner and Schekman, 2005). Many mitochondrial proteins or proteins destined for insertion into the endoplasmic reticulum (ER) carry *N*-terminal signals and often target the organelle of destination and translocate into or through the membrane in an unfolded state, while the majority of peroxisomal proteins display *C*-terminal extensions and are translocated in a folded, even multimeric or cofactor-bound state (Nyathi et al., 2013; Wiedemann and Pfanner, 2017; Walter and Erdmann, 2019; Kumar et al., 2024).

**Figure 1.**
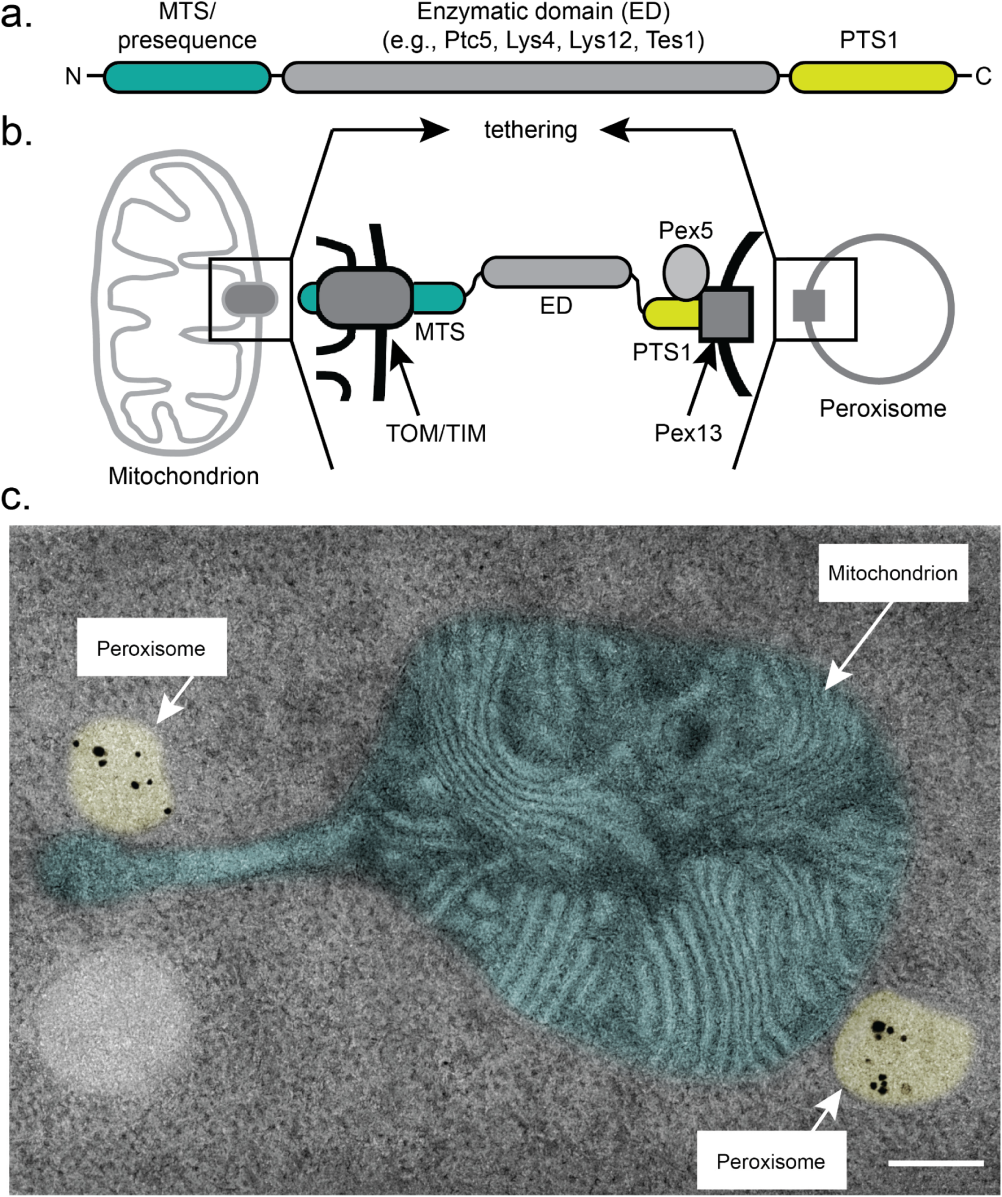
Dual targeting induced tethering. (a). Scheme for a typical MTS-PTS protein. These contain targeting signals at opposite termini and an enzymatic domain between these signals. More details about the enzymes can be found in Table 1. (b). Model highlighting tethering via dually targeted proteins. The mitochondrial translocation machinery (TOM/TIM; Wiedemann and Pfanner, 2017) is simplified. Pex5 and Pex13 are part of the transient peroxisomal import channel (Gao et al., 2023; Ravindran et al., 2023). (c). Transmission electron micrograph depicting contacts between peroxisomes (yellow) and mitochondria (magenta) induced by the overexpression of Ptc5 in *Ustilago maydis.* Immunogold labeling was performed to stain the peroxisomal marker protein mCherry-SKL (adapted from Bittner et al. 2024)*.* Scale bar: 0.2 µm.

However, many proteins are found in and execute functions in at least two organelles (Yogev and Pines, 2011; Bittner et al., 2022). Peroxisomes—tiny spherical organelles required for fatty acid breakdown—share several proteins for division and protein targeting with mitochondria—the major ATP-producing sites in cells (Costello et al., 2018; Bittner et al., 2022). Other proteins such as carnitine acetyl transferase also occur in both organelles to shuttle acetyl units (Elgersma et al., 1995; Ast et al., 2013). Dually localized proteins can possess targeting signals for both organelles for example, the protein phosphatase Ptc5 from *Saccharomyces cerevisiae* (from hereon called yeast) contains a mitochondrial targeting signal at its *N*-terminus and a *C*-terminal targeting signal for peroxisomes (Stehlik et al., 2020; Figure 1a). Ptc5 is dually localized in mitochondria and in peroxisomes. Interestingly, peroxisomal Ptc5 undergoes *N*-terminal truncation mediated by two mitochondrial peptidases, indicating that at least the very *N*-terminus has traversed both mitochondrial membranes before the processed polypeptide is targeted to peroxisomes (Stehlik et al., 2020). In peroxisomes, Ptc5 is responsible for the dephosphorylation of glycerol-phosphate dehydrogenase Gpd1, most likely to activate peroxisomal NAD+ regeneration necessary for β-oxidation (Jung et al., 2010; Al-Saryi et al., 2017; Stehlik et al., 2020). This function is corroborated by genetic data showing that deletion of *PTC5* leads to a synthetic growth defect in a strain lacking malate dehydrogenase Mdh3 involved in an alternative pathway for peroxisomal NAD+ regeneration (Hoppins et al., 2011; Costanzo et al., 2016; Al-Saryi et al., 2017; Bittner et al., 2022).

**Table 1.** Proteins identified in mitochondria that contain a PTS1.

*Saccharomyces cerevisiae*			
Standard name	Systematic name^a^	Function	References
CAT2	YML042W	Carnitine acetyl-CoA transferase	Elgersma et al., 1995; van Roermund et al., 1999
PTC5	YOR090C	Type 2C protein phosphatase	Vögtle et al., 2012; Stehlik et al., 2020
MRP7	YNL005C	Mitochondrial ribosomal protein	Fearon and Mason, 1988; Box et al., 2017
**TES1**	YJR019C	Peroxisomal acyl-CoA thioesterase	Jones et al., 1999
**MSS2**	YDL107W	Inner membrane protein of mitochondria	Simon et al., 1995; Broadley et al., 2001
CIT2	YCR005C	Peroxisomal citrate synthase	Lewin et al., 1990
PET309	YLR067C	Regulator of mitochondrial translation	Manthey and McEwen, 1995
LYS4	YDR234W	Homoaconitase	Wang et al., 1989
NSA1	YGL111W	Constituent of 66S pre-ribosomal particles	Harnpicharnchai et al., 2001
MIC10	YCL057C-A	Conserved component of the MICOS complex	Pfanner et al., 2014; Barbot et al., 2015
LYS12	YIL094C	NAD-linked homo-isocitrate dehydrogenase	Zabriskie and Jackson, 2000
**DPI8**	YJL133C-A	Putative mitochondrial protein of unknown function	Stehlik et al., 2020
CTA1	YDR256C	Peroxisomal catalase A	Cohen et al., 1985; Hiltunen et al., 2003
MRS1	YIR021W	Mitochondrial splicing protein	Kreike et al., 1986; Bassi et al., 2002
MRPL37	YBR268W	Mitochondrial ribosomal protein of the large subunit	Graack and Wittmann-Liebold, 1998
RML2	YEL050C	Mitochondrial ribosomal protein of the large subunit	Graack and Wittmann-Liebold, 1998
**PXP2**	YJR111C	Putative carboxymuconolactone decarboxylase	Nötzel et al., 2016; Stehlik et al., 2020
ATP8	Q0080	Subunit 8 of the F0 sector of mitochondrial ATP synthase	Devenish et al., 2000; Rak and Tzagoloff, 2009
***Ustilago maydis***			
**PTC5**	UMAG_01286^b^	Type 2C protein phosphatase	Bittner et al., 2024

a Gene ID according to *Saccharomyces* Genome Database;

b Gene ID according to National Center for Biotechnology Information; Bold letters indicate dual localization; green coloring indicates tethering function (Stehlik et al., 2020; Bittner et al., 2024).

In previous work several membrane proteins including the peroxisome biogenesis proteins Pex11 and Pex34, the subunit of the ER–mitochondria encounter structure ERMES complex protein Mdm34 and mitofusin Fzo1 were shown to contribute to peroxisome mitochondria (PerMit) contacts in yeast (Kornmann et al., 2009; Cohen et al., 2014; Mattiazzi-Ušaj et al., 2015; Shai et al., 2018; Castro et al., 2022; Alsayyah et al., 2024). Targeting of Ptc5 resembles a tug-of-war scenario, potentially fostering the formation of contact sites between both organelles as well (Figure 1a and 1b) as Ptc5 could attach to both organelles at the same time via interaction with the mitochondrial import complex and the peroxisomal targeting factor Pex5. Translocation of Ptc5 from mitochondria to peroxisomes might as well require organelle vicinity sites to occur.

## Decision for an Organelle: Translocation of Ptc5 Requires Contact Sites

In a high-throughput genetic screen for factors required for peroxisomal localization of Ptc5 in yeast, Mdm10 appeared as an interesting candidate—in Δ*mdm10* cells Ptc5 accumulates in mitochondria (Bittner et al., 2024). Mdm10 is a key protein of the ER-mitochondria encounter structure forming an interorganellar which contributes to lipid transfer from the ER to mitochondria (Kornmann et al., 2009; Wozny et al., 2023). It is also important for insertion of β-barrel proteins into mitochondria (Meisinger et al., 2004; Ellenrieder et al., 2016). It was suggested previously that ERMES connects peroxisomes to both of the other organelles resulting in a putative three-way junction (Cohen et al., 2014; Mattiazzi-Ušaj et al., 2015). Expression of a synthetic tether connecting peroxisomes and mitochondria enhances peroxisomal targeting of Ptc5 in ∆*mdm10* mutants demonstrating that loss of ERMES affects PerMit contacts and that these are required for translocation of Ptc5 (Bittner et al., 2024). It remains to be shown if ERMES tethers peroxisomes directly or if perturbation in mitochondrial protein import in ERMES mutants causes loss of contact and Ptc5 retention in mitochondria.

In a genetic screen in yeast designed to identify novel factors for the import of mitochondrial precursor proteins, the ER-localized DnaJ protein Djp1 was identified as an important factor (Hansen et al., 2018). Djp1 catalyzes the transit of precursors from the ER surface to mitochondria. An ER-mitochondria contact site formed by Djp1, the mitochondrial targeting factor Tom70 and the sterol transport protein Lam6 promotes the transit of precursors (Elbaz-Alon et al., 2015; Koch et al., 2024). This contact site was found to be partially redundant with ERMES with respect to mitochondrial targeting (Koch et al., 2024).

There are parallels between the findings discussed above and the work by Bittner et al., 2024. First, there is significant protein transit via the ER surface *en route* to mitochondria and via mitochondria *en route* to peroxisomes (Hansen et al., 2018; Stehlik et al., 2020; Bittner et al., 2024; Koch et al., 2024). Furthermore, both targeting pathways depend on organellar contact and even the machinery might overlap. ERMES was found to be important for both processes and Djp1 also belongs to the factors required for peroxisomal targeting of Ptc5 (Bittner et al., 2024; Koch et al., 2024). This adds to previous findings showing that mitochondria and peroxisomes share many proteins required for proper maintenance and proliferation (Costello et al., 2018; Bittner et al., 2022).

## Competing Targeting Signals: How Zip Codes Enable Organelle Tethering

Can proteins with targeting signals at opposite ends promote the formation of contact sites? For yeast Ptc5 this was not the case (Stehlik et al., 2020). Removal of the *N*-terminal transmembrane segment and of the cleavage site for the Inner Membrane Peptidase (IMP) complex (Nunnari et al., 1993), however, resulted in mitochondrial retention and efficient tethering upon overexpression (Stehlik et al., 2020).

Thus, proteins with competing targeting signals can act as tethers in principle and many of these are encoded in the yeast genome (Morgenstern et al., 2017; Stehlik et al., 2020). Overexpression of several increases the number of contacts between peroxisomes and mitochondria (Stehlik et al., 2020; Bittner et al., 2024; Figure 1b, Table 1); some clearly colocalize with both organelles, while others are mainly retained in mitochondria or predominantly accumulate in peroxisomes.

Remarkably, in the maize pathogenic fungus *Ustilago maydis* (Um) tethering occurs via an ortholog of Ptc5 (Figure 1c). Um_Ptc5 has an extended *N*-terminus compared to the yeast protein, is predominantly localized to mitochondria and might be no substrate for IMP (Stehlik et al., 2020; Bittner et al., 2024). Tethering is enhanced under conditions of increased peroxisome activity, for example, incubation in an oleic acid-supplemented medium coincides with the enrichment of Um_Ptc5 at PerMit contacts, which form more frequently under these conditions (Bittner et al., 2024).

Dual targeting signals at opposite termini are also contained in two enzymes involved in lysine biosynthesis in yeast—the lysine biosynthetic pathway occurs in four cellular compartments: nucleus, mitochondrion, cytosol and peroxisome; this compartmentalization is important for its correct function and regulation (Zabriskie and Jackson, 2000; Breitling et al., 2002; Al-Saryi et al., 2017; David et al., 2022). While the final step of lysine biosynthesis catalyzed by NAD+ requiring saccharopine dehydrogenase Lys1 takes place in peroxisomes of yeast and other fungi (Breitling et al., 2002; Al-Saryi et al., 2017), homoaconitase Lys4 and NAD linked homo-isocitrate dehydrogenase Lys12 both contain functional PTS1 motifs yet predominantly colocalize with mitochondria and act as tethers upon overexpression (Bittner et al., 2024; Figure 1 and Table 1). Upon induction of lysine biosynthesis in response to lysine limiting conditions enzymes participating in the pathway are induced and more connections between peroxisomes and mitochondria are formed. These connections require a functional PTS1 in Lys12 (Bittner et al., 2024). In the case of the Lys4 and Lys12 the PTS1 motifs appear to predominantly fulfill a tethering function, but do not mediate efficient translocation to the peroxisome—similar to Um_Ptc5, which does not translocate well but tethers well.

Efficient tethering appears to counteract removal from mitochondria and subsequent peroxisomal targeting (Bittner et al., 2024). This is true for proteins following the presequence pathway such as the Lys proteins, Ptc5 and to some extent carnitine acetyltransferase Cat2 from yeast. Another group of proteins with MTS and PTS1 for example, the putative peroxisomal thiolase Tes1 and the uncharacterized protein Pxp2 behave differently. Both do not follow the classical presequence pathway, are not processed at their *N*-termini, accumulate in foci at mitochondria and act as tethers for peroxisomes albeit they are not heavily pulled inside mitochondria (Bittner et al., 2024). Homogeneous mitochondrial localization of Pxp2 is only observed in cells lacking Pex5 but not in wild-type cells. The tethering executed by these proteins probably differs from the simplified scheme shown in Figure 1. They enrich in foci at mitochondria (maybe on the surface) but require the peroxisomal targeting factor Pex5 to promote tethering such as the other proteins discussed above (Bittner et al., 2024).

In conjunction, these findings point to a conserved second feature of targeting signals. They contribute to the formation of organelle contact sites beyond their primary function as determinants of subcellular localization. Thus, it is the protein production and sorting process itself that already fosters organelle collaboration. In mammalian cells, a related process was discovered: an isoform of acyl coenzyme A binding domain protein 2 (ACBD2), that contains an MTS and a PTS1 can contribute to the formation PerMit contacts (Fan et al., 2016).

A screening experiment in yeast revealed a somewhat similar phenomenon for a second pair of organelles (Eisenberg-Bord et al., 2021): the yeast protein Cnm1 (contact nucleus mitochondria 1) contains an *N*-terminal targeting signal for the nuclear envelope and a *C*-terminal targeting signal for mitochondria that interacts with the targeting factor Tom70. This domain architecture makes Cnm1 a tether protein probably involved in regulating phospholipid homeostasis (Eisenberg-Bord et al., 2021). Again the interaction with a targeting factor (Tom70) facilitates contact site formation comparable to the role of Pex5 at PerMit contacts (Bittner et al., 2024). The data on tethering of MTS-PTS proteins (Stehlik et al., 2020; Bittner et al., 2024), the dual function of ERMES components for protein import and tethering (Meisinger et al., 2004; Kornmann et al., 2009; Koch et al., 2024) and the role of Tom70 as a tether protein (Eisenberg-Bord et al., 2021; Koch et al., 2024) suggest that protein targeting and organelle tethering require overlapping machinery and may even be considered as a unit.

## Dually Targeted Membrane Proteins: Different but Tethers as well

A synthetic transmembrane protein containing an *N*-terminal targeting signal for the ER and a *C*-terminal PTS1 efficiently tethers peroxisomes to the ER (Stehlik et al., 2020). No such protein is encoded in the yeast genome. Interestingly, a previous screen utilizing overexpression of mCherry-tagged yeast proteins combined with a Split Venus reporter to monitor PerMit contacts has identified several dually targeted membrane proteins that enhance the PerMit reporter signal and thus, potentially affect contact (Shai et al., 2018). One such protein is the mitofusin homolog Fzo1 involved in catalyzing the fusion of mitochondria (Hermann et al., 1998; Rapaport et al., 1998). Although membrane fusion and contact site formation are distinct processes, both require tethering (Eisenberg-Bord et al., 2016) providing a mechanistic rationale for Fzo1-dependent formation of PerMit contacts (Shai et al., 2018). Previous work revealed ER-localized mitofusin 2 (MFN2) as a tether for mitochondria in human cells, a function that is enhanced by an ER-specific MFN2 variant derived from alternative splicing (de Brito and Scorrano, 2008; Naón et al., 2023). Recently, it was demonstrated that peroxisomal Fzo1 in yeast acts as a tether that connects peroxisomes to mitochondria via mitochondrial Fzo1 to facilitate mitochondrial fusion (Alsayyah et al., 2024). Other dually localized candidate proteins identified by Shai et al. (2018) include members of the shared division machinery of peroxisomes and mitochondria such as the tail-anchor protein Fis1 or the division factors Pex11 and Caf4 (Shai et al., 2018).

While the molecular basis for tethering via MFN2/Fzo1 is established—they bind mitofusin molecules at opposite organelles resulting in the formation of a bridge (de Brito and Scorrano, 2008; Alsayyah et al., 2024)—other dually targeted proteins probably tether via different interactions. One possibility is the interaction with targeting machinery at the opposite organelle similar to the MTS-PTS proteins or Cnm1 introduced in the previous chapter.

A number of peroxisomal membrane proteins are synthesized in the vicinity of the ER and may transit through the ER to peroxisomes (Hoepfner et al., 2005; Aranovich et al., 2014; Jan et al., 2014; Mast et al., 2016), others are synthesized directly at the peroxisome (Zipor et al., 2009; Dahan et al., 2022). In analogy to the dually targeted proteins shared by mitochondria and peroxisomes, ER targeting of *bona fide* peroxisomal membrane proteins may contribute to the formation of contacts. Indeed, the tail anchor protein Pex15 can posttranslationally enter the ER via the GET—(guided entry of tail anchor proteins)—complex, but also directly target peroxisomes or even target mitochondria in the absence of GET (Schuldiner et al., 2008; Bittner et al., 2024). It can leave the ER and target peroxisomes aided by the ATPase Spf1 and the targeting factor Pex19 (Dederer et al., 2019; Bittner et al., 2024). Overexpression of Pex15 supports the tethering of peroxisomes and the ER (Bittner et al., 2024). In yeast, disruption of the GET pathway leads to the formation of aberrant, small and highly mobile peroxisomes, a phenotype similar to cells lacking Pex30, a protein implicated in ER-peroxisome tethering (David et al., 2013; Joshi et al., 2016; Wu et al., 2020; Ferreira and Carvalho, 2021; Bittner et al., 2024). Simultaneous depletion of GET components and Pex30 leads to a severe growth defect and a significant reduction of functional peroxisomes that cluster at mitochondria (Bittner et al., 2024). This is in line with redundant mechanisms for ER–peroxisome tethering. Interestingly, an engineered variant of Pex15 targeted to mitochondria connects peroxisomes to this organelle (Bittner et al., 2024) suggesting that the subcellular distribution of dually targeted membrane proteins affects the pairing of organelles in a more general way. This can happen via homodimerization but likely via other interactions as well. Dually targeted proteins can even be considered as destined to stimulate tethering as they will almost certainly interact with proteins at both organelles—probably more transient in the case of MTS-PTS proteins and more stable in the case of membrane-anchored proteins.

## Conclusions and Perspectives

Most identified contact-forming proteins are membrane proteins or membrane-associated proteins that establish bridges between organelles, with regulation occurring, for example, through posttranslational modifications like phosphorylation (Scorrano et al., 2019; Di Mattia et al., 2020; Kors et al., 2022; Voeltz et al., 2024). The exploration of tethering events between organelles driven by dual protein targeting unveils a novel area for research (Stehlik et al., 2020; Bittner et al., 2024). To further explore this type of tethering the following questions are interesting to address.

1. What is exchanged at contact sites that are formed by MTS-PTS proteins?
2. Does this relate to the enzymatic functions of these proteins?
3. Can tethering *via* dually targeted membrane proteins support the maintenance of peroxisomes?
4. How significant is protein trafficking from the ER to peroxisomes, from the ER to mitochondria or from mitochondria to peroxisomes?
5. Does trafficking occur in each direction, for example, for tail anchor proteins?
6. What about other pairs of organelles?

Pursuing these questions will reveal the extent of protein trafficking between organelles and how this affects cellular organization and metabolism.
